# Differential Exposure to *Borrelia* spp. and Spotted Fever Group *Rickettsia* in Serbia and North Macedonia: A Comparative Study

**DOI:** 10.3390/pathogens14080814

**Published:** 2025-08-17

**Authors:** Dejan Jakimovski, Sofija Mateska, Marija Najdovska, Angela Stamenkovska, Verica Pavleva, Mile Bosilkovski, Dragana Mijatović, Verica Simin, Ivana Bogdan, Jasmina Grujić, Milica Simeunović, Miodrag Vranješ, Eleftherios Meletis, Polychronis Kostoulas, Olympia Lioupi, Pavle Banović

**Affiliations:** 1 Faculty of Medicine, Ss. Cyril and Methodius University in Skopje, 1000 Skopje, North Macedonia; 2 University Clinic for Infectious Diseases and Febrile Conditions, 1000 Skopje, North Macedonia; 3 Clinical Medicine Task Force, Balkan Association for Vector-Borne Diseases, 21000 Novi Sad, Serbia; 4 Public Health Institution General Hospital, 1480 Gevgelija, North Macedonia; 5 Infectious Disease Department, Clinical Hospital Shtip, 2000 Shtip, North Macedonia; 6 Department of Microbiology, Pasteur Institute Novi Sad, 21000 Novi Sad, Serbia; draganav77@gmail.com (D.M.);; 7 Diagnostics and Laboratory Research Task Force, Balkan Association for Vector-Borne Diseases, 21000 Novi Sad, Serbia; jasmina.grujic@mf.uns.ac.rs; 8 Department of Transfusiology, Faculty of Medicine in Novi Sad, University of Novi Sad, 21000 Novi Sad, Serbia; 9 Blood Transfusion Institute of Vojvodina, 21000 Novi Sad, Serbia; 10 Faculty of Medicine in Novi Sad, University of Novi Sad, 21000 Novi Sad, Serbia; 11 Faculty of Public and One Health, University of Thessaly, 43100 Karditsa, Greece; 12 Epidemiology and Biostatistics Research Task Force, Balkan Association for Vector-Borne Diseases, 21000 Novi Sad, Serbia; 13 Clinic for Lyme Borreliosis and Other Tick-Borne Diseases, Department of Prevention of Rabies and Other Infectious Diseases, Pasteur Institute Novi Sad, 21000 Novi Sad, Serbia; 14 Department of Microbiology with Parasitology and Immunology, Faculty of Medicine in Novi Sad, University of Novi Sad, 21000 Novi Sad, Serbia

**Keywords:** Balkans, *Borrelia*, North Macedonia, *Rickettsia*, Serbia, tick-borne diseases

## Abstract

Several diseases caused by tick-borne pathogens, including Lyme borreliosis (LB) and spotted fever group rickettsioses, are endemic in the Balkan Peninsula, positioned between Central Europe and the Middle East. This cross-sectional study aimed to assess serological exposure to *Borrelia* spp. and spotted fever group *Rickettsiae* (SFGR) among individuals with recent tick bites and healthy controls in two Balkan countries—Serbia and North Macedonia. Serum samples from 223 participants were tested for anti-*Borrelia* and anti-SFGR IgG antibodies. SFGR exposure was significantly higher in tick-exposed individuals from Skopje (North Macedonia) compared to those from Novi Sad (Serbia) (30.9% vs. 8.0%; *p* = 0.003). In contrast, anti-*Borrelia* IgG was more frequently detected in Novi Sad, though differences did not reach statistical significance. The findings support a north-to-south gradient in *Borrelia* exposure and a reverse trend for SFGR, consistent with earlier studies and regional tick infection data. Given the high SFGR exposure and limited clinical reporting in North Macedonia, the results highlight the likelihood that tick-borne rickettsioses remain under-recognized. Additionally, *Borrelia* exposure in North Macedonia warrants further investigation. These findings emphasize the need for enhanced tick-borne disease surveillance, identification of endemic zones, and improved diagnostic and public health infrastructure in both countries.

## 1. Introduction

The Balkan Peninsula lies at the crossroads between Central Europe and the Middle East, characterized by diverse geographic landscapes and transitions between temperate and subtropical climate zones [1]. Numerous Balkan regions have been identified as endemic for specific tick-borne diseases (TBDs), such as Lyme borreliosis (LB), tick-borne encephalitis, Crimean–Congo hemorrhagic fever, and rickettsioses [2,3,4,5,6,7]. These endemic areas are scattered throughout the peninsula, including Serbia and North Macedonia—neighboring countries that span across the western and central Balkans.

Recent studies conducted in Serbia and North Macedonia revealed a remarkably high prevalence of spotted fever group *Rickettsiae* (SFGR) in ticks removed from humans [1,5]. Although cases of tick-borne rickettsioses have been reported in both countries [3,8], these diseases may be considerably under-recognized, as no dedicated surveillance systems are currently in place. Awareness of the frequency of SFGR exposure is particularly important during the early stages of disease presentation, as timely recognition can guide clinical management [9,10]. In severe forms of tick-borne rickettsiosis, early administration of appropriate antibiotic therapy is critical, as delayed treatment has been associated with serious complications and, in some cases, fatal outcomes [11,12]. Therefore, improving clinical awareness and strengthening diagnostic and surveillance capacities are essential to reducing morbidity and mortality associated with this neglected but emerging disease.

LB is traditionally considered the most prevalent TBD in Europe, with over 120,000 cases reported annually; thus, its incidence shows temporal and spatial variation across the continent [13,14]. Detecting these fluctuations through surveillance systems is crucial, as it enables more efficient allocation of resources for the prevention and control of LB and other TBDs. Like most European countries, North Macedonia has implemented LB surveillance and lists it as a reportable disease [13,15]. However, the effectiveness of this system is questionable, with only four LB cases reported at the national level between 2021 and 2024 [15]. In contrast, Serbia discontinued LB surveillance in 2017, despite being considered endemic for the disease, as the presence of both tick vectors and pathogenic *Borrelia* species has been confirmed in multiple studies and case reports [5,16].

In the absence of reliable surveillance systems, population-based seroprevalence studies offer valuable insights into the true extent of exposure to tick-borne pathogens [17]. Such studies can reveal patterns of silent or under-reported transmission and help estimate the public health burden more accurately [17]. Therefore, the aim of the present study is to assess the frequency of exposure to these pathogens in two distinct groups: individuals with recent tick infestations and healthy control participants representing the general population. This approach seeks to inform future risk assessment, clinical awareness, and public health planning in Serbia and North Macedonia.

## 2. Materials and Methods

### 2.1. Study Framework and Recruitment of Participants

This cross-regional retrospective study was carried out in Skopje (North Macedonia) and Novi Sad (Serbia), and included patients who had sought medical attention for tick infestation at two collaborating institutions: Clinic for Infectious Diseases in Skopje (CIDS) and Pasteur Institute in Novi Sad (PINS). Samples were collected during the period of January–December 2023. Individuals were invited to take part in the study four weeks after tick removal, allowing time for potential development of seroconversion and detectable immune response. Blood samples from patients who consented to participate were collected at their respective institutions between four weeks and six months following the tick bite. For each center, a control group was created, consisting of healthy people who lived in the same administrative area (i.e., City of Skopje and City of Novi Sad) and had no recent tick infestation nor history related to TBDs. Participants’ demographic information, such as age and sex, was documented. For clarity, in the following sections, participants are referred to as “Tick-exposed cohort Skopje” and “Healthy donors Skopje” for individuals enrolled through CIDS, and as “Tick-exposed cohort Novi Sad” and “Healthy donors Novi Sad” for those recruited via PINS.

### 2.2. Laboratory Analysis

After acquiring written informed consent, venous blood samples (3 mL) were collected from each participant using BD Vacutainer^®^ SST™ tubes (BD, Franklin Lakes, NJ, USA). After collection, tubes were kept at room temperature to allow clot formation, followed by centrifugation at 2000× *g* for 10 min to separate the serum. The obtained serum samples were heat-inactivated at 56 °C for 30 min and subsequently stored at −80 °C until further analysis at the PINS.

### 2.3. Detection of Anti-Borrelia spp. IgG

Serum samples were analyzed for the presence of IgG antibodies against *Borrelia burgdorferi* sensu lato (s.l.) using a commercial enzyme-linked immunosorbent assay (ELISA) kit (recomWell *Borrelia* IgG, Mikrogen Diagnostik GmbH, Neuried, Germany; Cat. No. 4204). The assay utilizes recombinant OspC and VlsE antigens derived from *B. burgdorferi* sensu stricto (s.s.), *Borrelia garinii*, and *Borrelia afzelii*. All procedures were performed according to the manufacturer’s protocol, including the use of positive, negative, and cut-off controls provided with the kit. Results were interpreted qualitatively. Optical density (O.D.) was measured at 450 nm using an ELISA reader (ELX800, BioTek Instruments, Winooski, VT, USA). Samples with values ≥ 24 units/mL were considered positive, while values < 24 units/mL were classified as negative, in accordance with the manufacturer’s criteria.

### 2.4. Detection of Anti-SFGR IgG Antibodies

Serological detection of IgG antibodies reactive to *Rickettsia* spp. was performed using a commercial ELISA kit (Vircell S.L., Granada, Spain; Cat. No. G/M1005), with microplates coated with cells containing *Rickettsia conorii* as the representative SFGR antigen. The assay was carried out according to the manufacturer’s instructions. Results were interpreted qualitatively by calculating an antibody index (AI), derived by dividing the O.D. of the test sample by the mean O.D. of the cut-off control serum and then multiplying the result by 10. An AI value below 11 was considered negative, while values above 11 were interpreted as positive. O.D. was measured at 450 nm using the same ELISA reader (ELX800). Due to known antigenic cross-reactivity among SFGR members, the assay was used to assess genus-level exposure without species-specific discrimination.

### 2.5. Statistical Analysis

Descriptive statistics were used to summarize the demographic characteristics of the study population. Seroprevalence rates of IgG against *Borrelia* and SFGR antigens were calculated for each group and stratified by study site, exposure status, sex, and age category. The age groups were categorized as follows: young adults (18–30 years), middle-aged adults (31–50 years), and older adults (51–65 years). To assess the associations between seropositivity and selected risk factors, relative risk (RR) values were calculated. Comparisons between categorical variables were conducted using the Chi-square *(χ*^2^*)* test with Yates’s correction, or Fisher’s exact test when expected frequencies in any cell were below five. Statistical significance was defined as a *p*-value less than 0.05. All analyses were performed using GraphPad Prism version 9.0 (GraphPad Software, La Jolla, CA, USA).

## 3. Results

### 3.1. Participant Characteristics and Cohort Distribution

During the year 2023, a total of 492 and 530 patients presented for consultation to CIDS and PINS, from which 68 and 50 participants were enrolled, respectively (Table 1). None of the enrolled participants developed clinical signs of LB or SFGR during the follow-up period. Control groups for both regions consisted of healthy donors from Skopje (*n* = 55) and Novi Sad (*n* = 50) with no recent history of tick exposure or a history involving symptoms of the TBDs studied (Table 1).

### 3.2. Comparative Analysis of Anti-SFGR IgG Seropositivity

**Figure 1:**
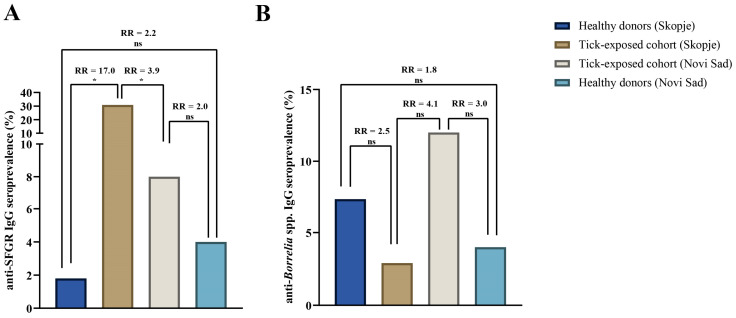
Seroprevalence of anti-SFGR IgG and anti-*Borrelia* IgG among tick-exposed and healthy donor groups in Skopje and Novi Sad: (**A**) anti-SFGR IgG seroprevalence by tick exposure and geographic location; (**B**) anti-*Borrelia* IgG seroprevalence by tick exposure and geographic location. RR: relative risk, indicating the probability of an individual in the studied groups being seropositive. The significance of the association was also tested by Fisher’s exact test; * statistically significant, *p* < 0.05; ns, non-significant, *p* > 0.05.

When seroprevalence values were compared by geographic region, tick-exposed individuals from the Skopje cohort showed almost four times higher exposure to SFGR compared to Novi Sad (30.9% [21/68] vs. 8.0% [4/50]; RR = 3.9, *p* = 0.003) (Figure 1A). Healthy participant comparison among cities did not reach statistical significance (*p* = 0.604); however, the Novi Sad healthy donor cohort showed a higher chance (RR = 2.2) of being exposed to SFGR compared to their Skopje counterparts (Figure 1A).

Among all individuals recruited in the Skopje region, tick-exposed individuals had a 17-fold higher relative risk of SFGR seropositivity compared to controls (30.9% [21/68] vs. 1.8% [1/55]; RR = 17.0; *p* < 0.00001). In contrast, the risk of SFGR exposure after a tick bite in the Novi Sad cohort was lower (RR = 2.0), without a statistically significant difference being observed between tick-exposed individuals and healthy donors (Figure 1A).

Although sex-stratified analysis did not reveal any statistically significant differences in seropositivity between the tick-exposed or non-exposed donors in either region, males from the Skopje tick-exposed group showed a slightly higher likelihood of seropositivity than females (34.2% [13/38] vs. 26.7% [8/30]; RR = 1.3, *p* = 0.504). Conversely, in the Novi Sad tick-exposed group, females were the ones who demonstrated a slightly higher risk of being seropositive (9.5% [2/21] vs. 6.9% [2/29]; RR = 1.4, *p* = 1.000) (Table 2).

No statistically significant differences in *Borrelia* and SFGR IgG seropositivity were observed when comparison was made across all age groups in both regions. Skopje tick-exposed group showed an age-associated rise in SFGR IgG seropositivity, with older adults exhibiting the highest rates and threefold risk to be seropositive when compared with young adults (32.5%, 13/40 vs. 10%, 1/10; RR = 3.3, *p* = 0.246). In contrast, the Novi Sad cohort seropositivity was confined predominantly to young adults (9.7%, 3/31), while no detectable levels of anti-SFGR IgG were found among older adults, suggesting the absence of a similar age-related trend.

### 3.3. Comparative Analysis of Anti-Borrelia IgG Seropositivity

Across the two study regions, tick-exposed individuals from Novi Sad showed four times higher risk for anti-*Borrelia* IgG seropositivity than those from Skopje (12.0% [6/50] vs. 2.9% [2/68]; RR = 4.1; *p* = 0.070), though this difference did not reach statistical significance (Figure 1B). Among healthy donors, the control group from Skopje had almost twice the risk of being seropositive to *Borrelia* spp. antigens than its Novi Sad counterpart, but again, without statistical significance (7.3% [4/55] vs. 4.0% [2/50]; RR = 1.8, *p* = 0.680) (Figure 1B).

Within the Novi Sad region, tick-exposed individuals had a threefold higher risk of exposure to *Borrelia* antigens (12.0% [6/50] vs. 4.0% [2/50]; RR = 3.0; *p* = 0.272). In Skopje, an opposite trend was observed, with seropositivity two times higher in controls than in tick-exposed individuals (7.3% [4/55] vs. 2.9% [2/68]; RR = 2.5; *p* = 0.406), though neither difference was statistically significant (Figure 1B).

Due to the low number of anti-*Borrelia* IgG-positive individuals, subgroup analyses were limited in interpretability. No significant sex-based differences were observed in either region, with comparable seropositivity rates between males and females (Table 2). Age-based comparisons were likewise inconclusive; in Skopje, only older adults tested positive, while in Novi Sad, seropositivity appeared in both older and young adults but not in middle-aged individuals, with no significant differences observed (14.3%, [2/14] vs. 12.9%, [4/31]; RR: 1.1, *p* = 1.000).

### 3.4. Comparative Analysis of Dual Seropositivity

**Figure 2:**
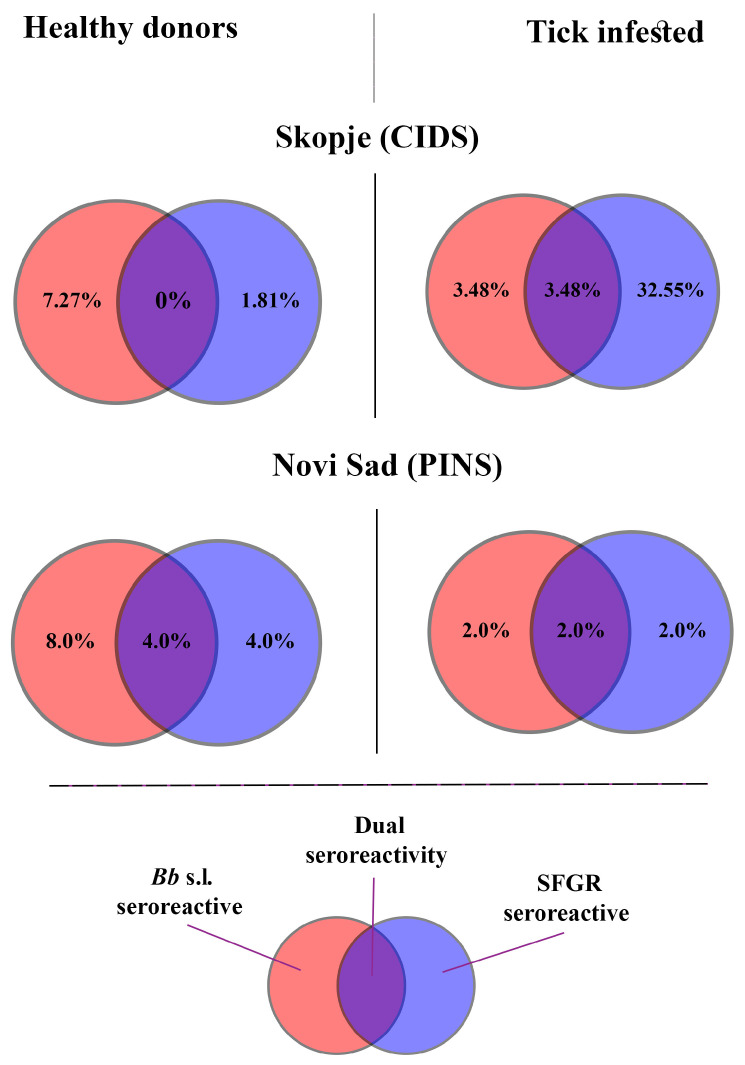
Single and dual exposure to examined tick-borne pathogens among tick-infested and healthy donor groups in Skopje (CIDS) and Novi Sad (PINS).

Across the two study regions, co-exposure to both pathogens was observed in all cohorts except in healthy donors from Skopje (3/4; 75%) (Figure 2). Among tick-infested individuals, dual exposure was most commonly observed in Skopje (3.48%). Surprisingly, the rate of dual exposure was highest in healthy donors from Novi Sad (4.0%) (Figure 2).

## 4. Discussion

An examination of anti-SFGR and anti-*Borrelia* IgG seroprevalence revealed significant differences in pathogen exposure risk between individuals from two neighboring Balkan countries. Specifically, tick infestation in Skopje was significantly associated with a higher likelihood of SFGR exposure compared to tick-infested individuals from Novi Sad, as well as compared to individuals from Skopje without prior tick exposure. Conversely, the likelihood of *Borrelia* spp. exposure among individuals with confirmed tick infestation was approximately four times higher in the Novi Sad cohort compared to the matched group in Skopje; however, this difference was not statistically significant.

The results of this study suggest the existence of a latitudinal gradient in *Borrelia* spp. and SFGR exposure across the Balkans, with a higher likelihood of *Borrelia* spp. exposure in the north (Novi Sad), decreasing toward the south (Skopje), where the prevalence of SFGR exposure increases.

Previous serosurveys also demonstrated a higher seroprevalence of anti-*Borrelia* IgG in the northern and central regions of the Balkans (e.g., Serbia [18] and Bulgaria [19]) compared to the southern regions (e.g., North Macedonia [18] and Greece [20]).

The results of our study are consistent with previous findings, where *Borrelia* spp. was detected in 8.12% of ticks removed from patients in Serbia, while it was not detected in ticks removed from individuals in North Macedonia. Similarly, cases of LB are more frequently reported in the northern and central Balkans (e.g., Serbia, Romania, and Bulgaria) [14], whereas the status of the disease in North Macedonia and Greece remains unclear, with only sporadic cases reported [18,20]. In contrast, SFGR members were detected in 44.89% of ticks removed from the Serbian population, while nearly all ticks (94.11%) removed from the Macedonian population were infected with at least one SFGR member [1].

Due to the lack of previous data on SFGR circulation in North Macedonia, it remains unclear whether the high prevalence of SFGR in ticks reflects the presence of long-established natural foci or the emergence of newly formed ones, as has been described in northern regions of Romania [21]. Further studies are needed to determine whether the detected latitudinal gradient in SFGR prevalence could possibly be associated with ecological and environmental factors such as climate, landscape type, and vector distribution [22,23,24].

While clinical SFGR infections in Serbia are predominantly described as atypical and mild [3,5], neighboring countries such as Romania and Bulgaria are reporting cases of moderate and severe Mediterranean spotted fever (MSF) [21,25], and clinical data on SFGR infections in the Macedonian population remain limited [8]. Considering the findings of frequent SFGR exposure and previously reported high *Rickettsia* spp. infection rate in ticks removed from humans [1], there is a high possibility that tick-borne rickettsioses are endemic in North Macedonia. The high level of SFGR exposure in the Macedonian population, paired with the absence of clinical manifestations among the examined individuals, may suggest circulation of low virulence *Rickettsia* spp. in the local tick population [26,27,28].

Notably, the age distribution of the tick-exposed cohorts differed between Skopje and Novi Sad, most likely reflecting center-specific recruitment and catchment demographics rather than a biological difference; accordingly, city-level contrasts were interpreted with caution and are presented as stratified by age. The relatively small sample size represents a clear limitation of this study, nevertheless, the results are of foundational importance for establishing future SFGR endemicity zones in North Macedonia. This is particularly relevant because in settings lacking laboratory diagnostic capacities, epidemiological criteria, when combined with clinical criteria, can be sufficient to establish a diagnosis of MSF [29]. Spatiotemporal models implemented within a Bayesian framework could incorporate findings from current and future seroprevalence studies as prior information to construct multi-parameter models for identifying high-risk areas for SFGR exposure within each country [30]. Additionally, the routine implementation of rickettsial diagnostics in patients hospitalized within 30 days of a tick bite would allow for the implementation of early warning tools for early detection of tick-borne rickettsiosis outbreaks [31]. This approach would support timely public health responses and inform the deployment of targeted diagnostic and therapeutic protocols. Additionally, the detection of anti-*Borrelia* IgG in the Macedonian cohort warrants larger, multicenter seroprevalence studies to assess regional risk levels for LB. To enable the timely detection and treatment of potential cases of both TBDs, it is essential to identify endemic zones in both countries and to establish a surveillance system that supports case reporting, clinical characterization, and effective communication pipelines.

## Figures and Tables

**Table 1:** Distribution of analyzed subjects according to risk groups in North Macedonia (Skopje) and Serbia (Novi Sad) (*n* = 223).

Risk Group	Tick-Exposed Cohort					Healthy Donors				
	Skopje		Novi Sad			Skopje		Novi Sad		
	*n*	(%)	*n*	(%)	*p*	*n*	(%)	*n*	(%)	*p*
Gender										
Male	38	(55.9)	29	(58)	0.818	25	(45.5)	27	(54)	0.382
Female	30	(44.1)	21	(42)		30	(54.5)	23	(46)	
Total	68	(100)	50	(100)	n/a	55	(100)	50	(100)	n/a
Age										
Young adults	10	(14.7)	31	(62)	0.000 * 0.026 * 0.001 *	27	(49.1)	22	(44)	0.601
Middle-aged adults	18	(26.5)	5	(10)		17	(30.9)	16	(32)	0.904
Older adults	40	(58.8)	14	(28)		11	(20)	12	(24)	0.621
Total	68	(100)	50	(100)	n/a	55	(100)	50	(100)	n/a

The *p*-values represent the results of two-sided comparisons of Skopje vs. Novi Sad within each subgroup of each cohort, calculated with the Chi-square *(χ*^2^*)* test with Yates’s correction. Row-wise *p*-values result from comparing the listed age category vs. all other ages between Skopje and Novi Sad within each cohort; * statistically significant, *p* < 0.05; n/a: not applicable.

**Table 2:** Seroprevalence of IgG antibodies against *Borrelia* and SFGR antigens, by center, tick exposure, and sex (CIDS = Clinic for Infectious Diseases, Skopje, North Macedonia; PINS = Pasteur Institute Novi Sad, Serbia) (*n* = 223).

Centre	Group	Sex	Samples Tested *n* (%)	Anti-SFGR IgG *n* (%)	*p*	Anti-*Borrelia* spp. IgG *n* (%)	*p*
CIDS	Tick-exposed	Male	38 (55.9)	13 (34.2)	0.504	1 (2.6)	1.000
		Female	30 (44.1)	8 (26.7)		1 (3.3)	
		Total	68 (100)	21 (30.9)	n/a	2 (2.9)	n/a
	Healthy donors	Male	25 (45.5)	0 (0.0)	1.000	2 (8.0)	1.000
		Female	30 (54.5)	1 (3.3)		2 (6.6)	
		Total	55 (100)	1 (1.8)	n/a	4 (7.3)	n/a
Total			123 (100)	22 (17.9)	n/a	6 (4.9)	n/a
PINS	Tick-exposed	Male	29 (58.0)	2 (6.9)	1.000	3 (10.3)	0.686
		Female	21 (42.0)	2 (9.5)		3 (14.3)	
		Total	50 (100)	4 (8.0)	n/a	6 (12.0)	n/a
	Healthy donors	Male	27 (54.0)	1 (3.7)	1.000	1 (3.7)	1.000
		Female	23 (46.0)	1 (4.3)		1 (4.3)	
		Total	50 (100)	2 (4.0)	n/a	2 (4.0)	n/a
Total			100 (100)	6 (6.0)	n/a	8 (8.0)	n/a

The *p*-values represent the results of the Chi-square *(χ*^2^*)* test with Yates’s correction (or Fisher’s exact test when any cell count < 5), comparing the proportion seropositive between males and females within the same center and exposure group. n/a: not applicable.

## Data Availability

All data generated in this manuscript are available in the main text.

## Abbreviations

The following abbreviations are used in this manuscript:

AI	Antibody index
CIDS	Clinic for Infectious Diseases in Skopje
ELISA	Enzyme-linked immunosorbent assay
LB	Lyme borreliosis
MSF	Mediterranean spotted fever
O.D.	Optical density
PINS	Pasteur Institute in Novi Sad
RR	Relative risk
SFGR	Spotted fever group *Rickettsia*
TBDs	Tick-borne diseases
